# Comparative sequence analysis of Solanum and Arabidopsis in a hot spot for pathogen resistance on potato chromosome V reveals a patchwork of conserved and rapidly evolving genome segments

**DOI:** 10.1186/1471-2164-8-112

**Published:** 2007-05-02

**Authors:** Agim Ballvora, Anika Jöcker, Prisca Viehöver, Hirofumi Ishihara, Jürgen Paal, Khalid Meksem, Rémy Bruggmann, Heiko Schoof, Bernd Weisshaar, Christiane Gebhardt

**Affiliations:** 1 Max-Planck Institut für Züchtungsforschung, Carl von Linné Weg 10, 50829 Köln, Germany; 2 Institut für Genomforschung, Universität Bielefeld, Universitätsstrasse 25, 33615 Bielefeld, Germany; 3 Southern Illinois University at Carbondale, Dept. of Plant, Soil and General Agriculture, Carbondale, IL62901-4415, USA; 4 GSF Forschungszentrum für Umwelt und Gesundheit, Institut für Bioinformatik, Ingolstädter Landstr. 1, 85764 Neuherberg, Germany

## Abstract

**Background:**

Quantitative phenotypic variation of agronomic characters in crop plants is controlled by environmental and genetic factors (quantitative trait loci = QTL). To understand the molecular basis of such QTL, the identification of the underlying genes is of primary interest and DNA sequence analysis of the genomic regions harboring QTL is a prerequisite for that. QTL mapping in potato (*Solanum tuberosum*) has identified a region on chromosome V tagged by DNA markers *GP21 *and *GP179*, which contains a number of important QTL, among others QTL for resistance to late blight caused by the oomycete *Phytophthora infestans *and to root cyst nematodes.

**Results:**

To obtain genomic sequence for the targeted region on chromosome V, two local BAC (bacterial artificial chromosome) contigs were constructed and sequenced, which corresponded to parts of the homologous chromosomes of the diploid, heterozygous genotype P6/210. Two contiguous sequences of 417,445 and 202,781 base pairs were assembled and annotated. Gene-by-gene co-linearity was disrupted by non-allelic insertions of retrotransposon elements, stretches of diverged intergenic sequences, differences in gene content and gene order. The latter was caused by inversion of a 70 kbp genomic fragment. These features were also found in comparison to orthologous sequence contigs from three homeologous chromosomes of *Solanum demissum*, a wild tuber bearing species. Functional annotation of the sequence identified 48 putative open reading frames (ORF) in one contig and 22 in the other, with an average of one ORF every 9 kbp. Ten ORFs were classified as resistance-gene-like, 11 as F-box-containing genes, 13 as transposable elements and three as transcription factors. Comparing potato to *Arabidopsis thaliana *annotated proteins revealed five micro-syntenic blocks of three to seven ORFs with *A. thaliana *chromosomes 1, 3 and 5.

**Conclusion:**

Comparative sequence analysis revealed highly conserved collinear regions that flank regions showing high variability and tandem duplicated genes. Sequence annotation revealed that the majority of the ORFs were members of multiple gene families. Comparing potato to *Arabidopsis thaliana *annotated proteins suggested fragmented structural conservation between these distantly related plant species.

## Background

The potato (*Solanum tuberosum*) is the most important crop of the Solanaceae. It is a tetraploid, non-inbred, annual plant species that is vegetatively propagated by tubers. Polyploidy and inbreeding depression prevent the generation of homozygous lines. When the ploidy level is reduced from 4n to 2n, the diploid potatoes are self incompatible. Potato genotypes at all ploidy levels are therefore heterozygous [1]. The basic chromosome number of potato is twelve and its genome size is in the order of 800 to 1000 megabases, similar to the closely related tomato (*Solanum lycopersicum*). Detailed RFLP (restriction fragment length polymorphism) linkage maps have been constructed for the twelve chromosomes [2-5,63], which were subsequently used to locate in the potato genome factors controlling monogenic and polygenic traits of agronomic relevance such as resistance to pests and pathogens or tuber quality (e. g. starch and sugar content) (reviewed in [1,6]). When using the same locus specific DNA-based markers in different mapping populations, the positional information of the mapped factors controlling qualitative and quantitative traits can be compared and integrated. This comparison showed that a number of the factors which control qualitative (*R *genes) or quantitative resistance (QRL = quantitative resistance loci) to different types of pathogens map to similar positions. These chromosomal regions are so-called hot-spots for pathogen resistance. One of the most conspicuous resistance hot-spots in the potato genome is located on potato chromosome V, in a chromosome segment tagged by the DNA-based markers *GP21 *and *GP179*. The 3 cM interval between *GP21 *and *GP179 *[7] includes the *R *genes *Rx2 *and *Nb *both for resistance to *Potato Virus X *[8,9] and the *R1 *gene for race-specific resistance to the oomycete *Phytophthora infestans *causing late blight [10,7]. The same markers are also linked to QRL for *P. infestans *[11-14] and QRL for the root cyst nematodes *G. rostochiensis and G. pallida *[15-18]. As shown by QTL mapping [12-14,19,20], this region on potato chromosome V not only contains genes for resistance to various pathogens but also genes controlling plant vigor, plant maturity (the time the plant needs from planting to reach maturity under long day conditions), tuber yield, tuber starch and tuber sugar content.

Two *R *genes from the chromosome V resistance hot-spot have been functionally characterized, *Rx2 *for extreme resistance to *Potato Virus X *[21] and *R1 *for resistance to *P. infestans *[22]. Both *R *genes are members of the superfamily of plant resistance genes characterized by a coiled coil (CC), a nucleotide binding (NB) and a leucine rich repeat (LRR) domain [23], but otherwise share low sequence similarity. *R1 *has been introgressed from the allo-hexaploid – wild potato species *Solanum demissum *into the cultivated potato germplasm pool [1,25] and is one member of a clustered gene family in the *GP21–GP179 *interval [22,25]. The molecular basis of the QRL for late blight and root cyst nematodes in the same region is unknown. One possibility is that alleles of the *R1 *and/or the *Rx2 *gene, or other members of the *R1 *gene family and/or another resistance-gene-like (RGL) family in this genome region encode the factors for the quantitative resistance phenotypes, similar to classical plant genetic studies, where resistance loci with multiple specificities to different races of a pathogen may be alleles of the same gene, or tightly linked genes [11,26]. However, the resolution of QTL mapping in this region of the potato genome, even when based on large populations related by descent [27] is low. Genes physically linked to the *R1 *family, but structurally and functionally unrelated to *R1 *or other RGLs, cannot be excluded as candidates for the QRL of interest. High resolution mapping and map-based cloning of QTL based on recombinant inbred populations or near isogenic lines [28] is not feasible in potato due to self-incompatibility. As an alternative approach, genomic sequencing and annotation can provide information on all putative genes present in the whole region, which then might be further examined for function as quantitative trait loci, first *in silico *by functional annotation and then experimentally by analysis of natural allelic diversity of positional candidates and complementation analysis using candidate gene allelic variants [28]. Powerful bioinformatic tools for gene annotation [29] and functional analysis of sequence related genes in model plants such as *Arabidopsis thaliana *and rice can facilitate the selection of functional candidate genes among all the positional candidates in a genome segment harboring QTL.

Parts of the genomic region corresponding to the *GP21–GP179 *interval in *S. demissum *were sequenced and three different haplotypes A, B and C equivalent to the three homeologous chromosome pairs of *S. demissum *were identified [25]. They demonstrated substantial structural variation among the haplotype sequences. In this paper, we report the genomic sequence analysis of two orthologous chromosome segments of the heterozygous, diploid *Solanum tuberosum *genotype P6/210 in the same *GP21–GP179 *interval. Two independent bacterial artificial chromosome (BAC) contigs, corresponding to the homologous chromosomes of P6/210 were constructed, sequenced and annotated, thereby extending the genomic sequence information available in this functionally important region of the potato genome. Comparative sequence analysis revealed regions with severe structural distortions and deviations from gene by gene co-linearity, which are flanked by conserved regions showing microsynteny with the *A. thaliana *genome.

## Results

### Contig construction and BAC sequencing

The potato clone P6/210 used to construct the BAC libraries was heterozygous for *R1 *(*R1/r1*). Physical mapping in the context of cloning the *R1 *resistance gene had identified overlapping BAC insertions that originated from the homologous chromosomes carrying either the *R1 *resistance allele or an *r1 *allele for susceptibility [22]. In order to obtain two contigs, one for each homologous chromosome in the *R1 *genomic region, the physical map was extended. Subsequently, we refer to the two contigs as the R1-contig and the r1-contig.

**Figure 1:**
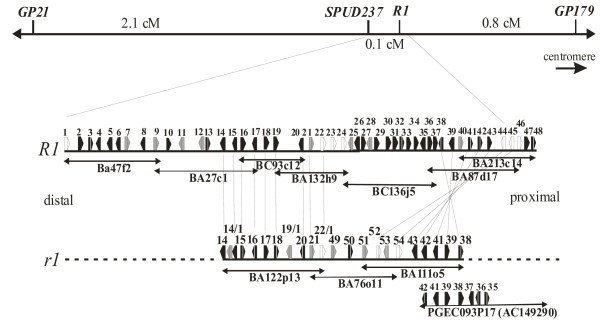
Physical map and position of the R1- and r1-contig in the genetic interval *GP21–GP179 *on potato chromosome V and their annotated gene content. Annotated ORFs are shown as rectangles with arrowheads indicating the direction of transcription, and are numbered from 1 to 54 according to Table 2. ORFs showing sequence homology to RGLs are shown in white, putative polyproteins and transposon-like ORFs are shown in gray and all other gene models in black. Putative orthologs between the R1 and r1 contig are connected by thin lines. More detailed descriptions of the ORF annotation is provided in Additional file 5.

The R1-contig was extended proximally, starting from BA27c1 by clone BC93c12 (Figure 1). This BAC was identified by PCR screening of the BC library using specific primers that were developed based on the sequence of the proximal BAC end of BA27c1. Additional 34 BACs from this region, all containing one or more members of the *R1 *gene family, were identified by colony filter hybridization using as probe a 1.4 kb DNA fragment amplified with *R1 *gene specific oligonucleotides 76-2sf2 and 76-2SR [22]. Among the clones containing *R1 *homologous genes were BA132h9 and BA213c14. Clone BA132h9 overlaps with BC93c12, and BA213c14 overlaps with BA87d17 (Figure 1). The gap between BA132h9 and BA87d17 was bridged by clone BC136j5 (Figure 1). From the seven BAC clones that constitute a minimal tiling path including the *R1 *locus, six BAC inserts were fully and one (BC93c12) was partially sequenced (Table 1). The r1-contig consisted of clones BA122p13 and BA76o11 and was extended proximally by clone BA111o5 (Figure 1). All three clones were fully sequenced (Table 1).

**Table 1:** Summary of sequenced BAC insertions of genotype P6/210.

BAC clone	Homologue	Sequence length [bp]
BA47f2	R1	81,720
BA27c1	R1	91,301
BC93c12	R1	6,826
BA132h9	R1	74,715
BC136j5	R1	101,140
BA87d17	R1	75,612
BA213c14	R1	72,505
BA122p13	r1	91,226
BA76o11	r1	71,514
BA111o5	r1	76,593

### Genomic sequence analysis

The 620, 226 kbp genomic sequence obtained from seven R1- and three r1-BAC insertions was assembled into two distinguished, unambiguous stretches of DNA sequence corresponding to the R1- and r1-contig of 417,445 base pairs [GenBank:EF514212] and 202,781 base pairs [GenBank:EF514213], respectively, using MegaMerger. Overall GC content in the R1- and r1-contig is 33.26% and 34.11%, respectively.

**Figure 2:**
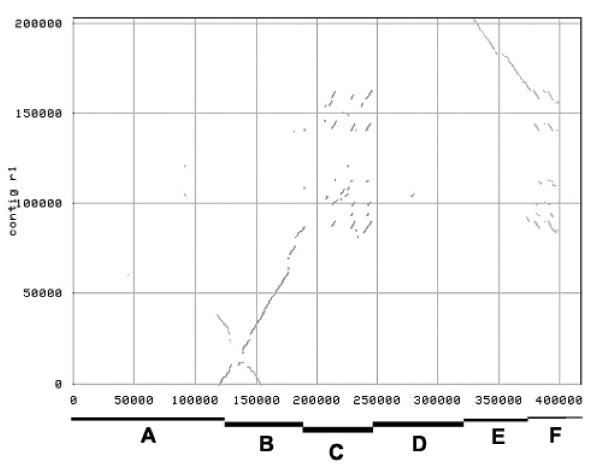
Dot-Plot comparison done using MUMmer of the DNA sequences of the R1- and r1-contig. Alignment between contigs starts at approximately 120 kbp in the R1-contig. The DNA sequence between approximately 120 kbp and 200 kbp of the R1-contig (B) was highly similar to the r1-contig and included a palindromic structure of around 34 kbp. The regions from 200 kbp to 245 kbp (C) and again from 375 to 400 kbp (F) of the R1-contig contained conserved repeated sequences that were interrupted and flanked by sequences with low similarity between the two contigs. Segment D from 245 to 330 kb showed no similarity to the r1-contig. The segment from 330 kbp to 400 kbp (E) of the R1-contig was inverted relative to the r1-contig.

The two contig sequences share highly similar and collinear regions, but these are interrupted by more variable regions (Figure 2). In addition, an inversion and an inverted repeat were identified. Tandem repeats corresponding to six copies of *R1 *homologous genes in the R1 contig and three copies in the r1 contig were identified. These repeats are embedded in a region of non-alignable sequence.

For easier reference, we label regions along the R1 contig from A to F (Figure 2) based on features revealed by comparison of the R1 with the r1 contig using MUMmer (Figure 2). For region A, no sequence was obtained from r1. Region B is highly similar to the start of contig r1. In region C, co-linearity and alignment are disturbed, and similarity is primarily detected in three tandem repeats. No similarity to r1 is detected in region D. Region E (69,850 bp) again aligns well with r1, but in reverse orientation, indicating a genomic inversion. Region F, resembling region C, is not aligned well but contains two tandem repeats that are highly similar to the tandem repeats in region C, but in inverse orientation. Region B contains a palindromic structure discussed in more detail below.

Comparison to the A, B and C haplotypes of the orthologous genome region in *Solanum demissum *[25] revealed similar features (supplementary Fig. S3 and S4). The R1 contig is most closely related to the A sequence, with co-linearity and high sequence identity (99%). However, we orient BAC PGEC472P22 (accession AC151815) of A in reverse orientation compared to [25], thereby introducing the inversion of A and R1 relative to r1, B and C (see Discussion). The r1 contig is more similar to the sequences of B and C haplotypes of *S. demissum*.

Regions A and B show co-linearity with the *S. demissum *genomic regions II and III as defined by another study [25], whereas regions C through F correspond to *S. demissum *genomic regions IV and V.

### Gene content

We predict that the R1 contig contains 48 genes, seven of which are transposons and three are pseudogenes. Thirteen protein coding genes on the r1 contig can be identified as orthologs of collinear genes on R1. However, on R1 there are two additional protein coding genes. Six transposon genes are specific to r1, and for one pseudogene, we could not identify a partner on R1. On average, one ORF is annotated in every 9 kbp of genomic sequence. The average GC content of exons is 39%.

A putative function was assigned based on similarity to proteins of known function with an E-value smaller than 10^-10 ^(Table 2, details in Additional file 5). Thirteen genes are related to retrotransposons. Two multigene families, F-box proteins and NBS (NBS-LRR) type resistance genes, are represented by several members. Six proteins on contig R1 and three on r1 are members of the *R1 *gene family. At least eleven F-Box proteins are found on R1, but none of these are found in r1-contig.

**Table 2:** Genomic sequence annotation of the R1- and r1-contig.

ORF No. ^(1)^	Manual Functional Annotation ^(2)^	ORF No.^(1)^	Manual Functional Annotation ^(2)^
1	Fragment of disease resistance protein	27	F-box protein-like
2	ZF-HD homeobox protein	28	MuDR transposase
3	Unknown protein	29	F-box protein
4	Ribosomal protein L34e	30	F-box protein
5	No apical meristem (NAM)-like	31	F-box protein
6	Hypothetical protein	32	F-box protein
7	Retrotransposon RNAse containing	33	F-box protein
8	Hypothetical protein	34	F-box associated domain containing protein
9	Retrotransposon	35	Hypothetical protein
10	F-box protein	36	Pseudogene (F-box protein-like)
11	Retrotransposon	37	F-box protein
12	RNAseH containing reverse transcriptase	38	TCP transcription factor
13	F-box protein	39	Unknown protein
14	RNA dependent RNA polymerase	40	Retrotransposon
14/1	Transposon fragment	41	Unknown protein
15	Hypothetical protein (Pseudogene)	42	Origin recognition complex subunit 6
16	RNA dependent RNA polymerase	43	Sterol desaturase/Acid phosphatase
17	CAAX amino terminal protease	44	Disease resistance protein (R1)
18	Methyltransferase	45	Disease resistance protein (R1_1, 82% identity to R1)
19	Phytochrome kinase substrate	46	Disease resistance protein (R1_2, 49.3% identity to R1)
19/1	Retrotransposon	47	HVA22-like protein
20	AAA ATPase	48	Regulator of chromosome condensation, RCC1
21	Retrotransposon(Pseudogene)	49	Retrotransposon
22	Disease resistance protein (R1-3, 74.7% identity to R1)	50	Hypothetical protein (Pseudogene)
22/1	Disease resistance protein (R1-6, 83.5% identity to R1)	51	Transposon-like
23	Disease resistance protein (R1_4, 80.2% identity to R1)	52	Disease resistance protein (R1_7, 80.7% identity to R1)
24	Disease resistance protein (R1_5, 85.9% to R1)	53	Retrotransposon
25	Retrotransposon	54	Disease resistance protein (R1_8, 83.2% identity to R1)
26	F-box protein-like		

^(1)^ORFs are numbered according to Figure 1.

^(2)^Manual functional annotation was based on BLAST hits and/or domain prediction results, see Methods. More details are shown in Additional file 5.

Where sequence from both contigs was available, most proteins and some transposon-related genes are conserved between the R1 and r1 contig (Figure 1 and Additional file 5). Notable exceptions are 13 retroelements, six of which were found only in the r1 contig (ORF 14/1, ORF 19/1, ORF 21, ORF 49, ORF 51 and ORF 53) and 7 in the R1 contig (ORF 7, ORF 9, ORF 11, ORF 12, ORF 25, ORF 28 and ORF 40). The palindromic structure in region B is formed by an inverted repeat of two highly similar RNA-directed RNA polymerases (ORFs 14 and 16), separated by a hypothetical protein (ORF 15). The r1 specific retrotransposon 14/1 is inserted between the inverted repeats. Region E contains five genes that are conserved between R1 and r1 but in reverse order and orientation, indicating a genomic inversion. The proximal two genes are members of the *R1 *family, ORF 44 being the functional *R1 *resistance gene (accession AF447489) and the following ORF 45 is a tandem duplicate. ORFs 44 and 45 are conserved in sequence, but having inverse order and orientation, with resistance gene homologues 52 and 54 on contig r1, suggesting that the inversion includes these two genes. However, proteins 44 and 45 are more similar to each other than to 52 or 54, indicating that they may have arisen through tandem duplication after the inversion event. Another resistance gene homologue (ORF 46) follows on contig R1, but is less similar to the other *R1*-related genes. Also two genes (ORFs 47 and 48) could not be related to any locus on contig r1. This suggests that the proximal breakpoint of the genomic inversion in contig R1 is after gene 44 or 45.

To map the distal breakpoint of the inversion event on contig R1, we used sequence from *S. demissum *BAC PGEC093P17 (accession AC149290), as the sequence of contig r1 did not extend sufficiently far in proximal direction. The PGEC093P17 sequence contains orthologs of proteins 43, 42, 41, 39 and 38 in the same order and orientation as found on contig r1, therefore inverted with respect to R1. As in r1, the R1 specific transposon ORF 40 is not found in PGEC093P17 (Fig. 1). Proximal to protein 38 followed probable orthologs of proteins 37, 36 and 35 in reverse order and orientation compared to contig R1, indicating that these three genes are part of the inversion. The remaining PGEC093P17 sequence proximal to ORF 35 contains only transposon fragments and hypothetical proteins, which are unrelated to genes distal to ORF 35 (mainly F-box genes, Table 2) in the R1-contig. This maps the distal inversion breakpoint in contig R1 between genes 34 and 35. As consequence of the inversion, genes 23 to 34 in the R1 contig are probably part of a region that is, at least at this genomic location, R1 specific. This region includes two *R1 *homologous genes (ORF 23 and 24) in tandem orientation to ORF 22, two transposon-related genes and a series of eight F-box genes. For the flanking regions of contig R1, we found almost perfect gene-by-gene co-linearity to r1 or PGEC093P17. We could not assign three genes in contig r1 to any collinear region, two of which are transposons and one resembles a fragmented resistance gene.

### Microsynteny with the *A. thaliana *genome

Five microsyntenic blocks were identified in *A. thaliana *based on the arbitrary criterion of finding at least three pairs of homologous protein sequences within a comparable physical distance (Table 3 and Additional file 6). When the potato annotated proteins were compared to the *A. thaliana *annotated proteins, only ORFs 6, 8, 15 and 35 did not have any hit. Each member of the 'resistance-gene-like' and 'F-box containing' gene families had more than 30 hits. Due to the high copy number, these two multigene families and the retrotransposons were not considered when searching for groups of in both species physically closely linked homologue pairs (microsynteny). The 26 remaining potato ORFs used for microsynteny analysis had on average 9.2 hits in the *A. thaliana *genome. The five syntenic blocks involve three sections within the potato contigs, (i) the distal ORFs 2, 3, 4 and 5, (ii) ORFs 17, 18 and 19 in the middle part and (iii) ORFs 38 to 48 in the proximal region. The syntenic blocks I, II and III comprise between 215 kbp and 405 kbp potato sequence and are syntenic with three different, smaller *A. thaliana *genome fragments from 7 kbp to 54 kbp on chromosome 1. The four consecutive *A. thaliana *genes in block I have the same relative orientation of transcription and the same order as the homologous potato ORF 17, 19, 43 and 41 in the r1-contig (Table 3, Figure 1). The largest syntenic region identified in both species is block III, including 7 potato ORFs, which spans nearly the whole R1-contig (405 kbp). The corresponding *A. thaliana *genome fragment is 54 kbp in size and contains 12 annotated genes, seven in the same order and homologous to ORFs in the r1-contig, one F-box gene (At1G69630.1) and four other, consecutive genes that are not syntenic (AtG69650.1 to AtG69680.1). Block II with four syntenic ORFs is second in size (345 kbp). The related *A. thaliana *genome fragment of only 18 kbp contain five annotated genes, four homologous to the potato ORFs and the fifth one different (AtG26860.1). Potato ORFs 2, 4 and 5 (block IV) and 2, 3 and 5 (block V) are located within 25 kbp in the R1-contig. The *A. thaliana *genome fragment IV on chromosome 3 is larger (106 kbp), whereas fragment V on chromosome 5 is nearly identical in size (28 kbp). The three ORFs in block V are not only located in genomic fragments of similar size in both species but also in the same order and in the same orientation of transcription. It is unlikely that the five syntenic blocks occur by chance only. We estimate the probability of finding three hits within 100 kb in *A. thaliana *to be less than 10^-3 ^using an approximation described in [4]: h^n ^× (0.1 Mbp/121 Mbp)^n-1^, where h is the average number of hits of the potato genes in *A. thaliana *(9.2), n the number of genes conserved in the syntenic block (3), 0.1 Mbp the size of the syntenic block in *A. thaliana *and 121 Mbp the size of the *A. thaliana *genome (giving 5 × 10^-4 ^for three genes with an average of 9.2 hits). None of the syntenic *A. thaliana *genome fragments include a putative or functional gene for pathogen resistance and only one includes an F-box containing gene. The *A. thaliana *gene *RPP13 *conferring resistance to *Peronospora parasitica *and having the highest sequence similarity to the *R1 *gene family (Additional file 5) is located on chromosome 3 outside any putatively syntenic region.

**Table 3:** Syntenic blocks between potato annotated genes in the R1- and r1-contigs and sequence related genes of *A. thaliana*.

Syntenic block	*S. tuberosum *ORF	*A. thaliana *ORF ^(1)^	*A. thaliana *BAC	*A. thaliana *ORF position ^(1) ^[Mbp]	*A. thaliana *block size	*S. tuberosum *block size
I	ORF17	At1G14270.1	F14L17	4.875	7 kbp	215 kbp
	ORF19	At1G14280.1	F14L17	4.878		
	ORF43	At1G14290.1	F14L17	4.880		
	ORF41	At1G14300.1	F14L17	4.882		
II	ORF4	At1G26880.1	T2P11	9.316	18 kbp	345 kbp
	ORF5	At1G26870.1	T2P11	9.313		
	ORF18	At1G26850.1	T2P11	9.301		
	ORF42	At1G26840.1	T2P11	9.298		
III	ORF2	At1G69600.1	F24J1	26.168	54 kbp	405 kbp
	ORF3	At1G69610.1	T6C23	26.190		
	ORF4	At1G69620.1	T6C23	26.193		
	ORF43	At1G69640.1	T6C23	26.197		
	ORF38	At1G69690.1	T6C23	26.221		
	ORF47	At1G69700.1	T6C23	26.224		
	ORF48	At1G69710.1	T6C23	26.226		
IV	ORF2	At3G28920.1	MYI13	10.941	106 kbp	25 kbp
	ORF4	At3G28900.1	K5K13	10.904		
	ORF5	At3G29035.1	K5K13	11.035		
V	ORF2	At5G39760.1	MKM21	15.928	28 kbp	25 kbp
	ORF3	At5G39785.1	MKM21	15.946		
	ORF5	At5G39820.1	MKM21	15.956		

^(1)^*A. thaliana *gene identifiers and position coordinates on the AGI physical map in Megabasesare according to TAIR release 6.

## Discussion

### Genome structure

We found extensive co-linearity of protein-coding genes, interrupted by unilateral insertions of retrotransposons and a region of highly diverged DNA sequence in the vicinity of clusters of tandem duplicated genes. This corresponds to findings in the orthologous genomic region of hexaploid *S. demissum *[25]. We show that a 70 kb region containing ten protein-coding genes is inverted in both the R1 contig and presumably also the A haplotype of *S. demissum*. While sequence similarity at the nucleotide level was not sufficient to precisely map the inversion breakpoints in the intergenic regions, order and orientation of homologous gene pairs were consistent without exception and allowed to estimate the position of the inversion breakpoints. This inversion is not evident in the available sequence of the *S. demissum *A haplotype, as BAC PGEC472P22 (accession AC151815) mainly contains genes from the inversion and only few beyond the inversion breakpoint. The gap in the sequence of haplotype A presumably led to BAC PGEC472P22 being oriented to achieve co-linearity with B and C haplotypes. Kuang et al. [25] do not present any data, e.g. mapping of BAC ends or overlaps with neighboring BAC clones, to verify the orientation. Thus, we take the high sequence similarity, uninterrupted across the presumed inversion break point, between the R1 contig and PGEC472P22 to indicate that the inversion also exists in the A haplotype (Additional files 3 and 4).

Currently, there are only few examples where comparative structural analysis of orthologous genome segments was performed in crop plants over several hundred kb. In all cases reported, micro-structural diversity was found, e.g. in *Zea mays *[30,31]. In maize the major contribution to diversity stems from LTR retrotransposons. In our analysis transposon related genes are less conserved, also suggesting recent insertions and deletions. In tomato (*S. lycopersicum*), a segment on chromosome 6 containing the *Mi-1 *gene for resistance to root knot nematodes, which has been introgressed from *S. peruvianum*, was shown to be inverted in resistant when compared to susceptible tomato genotypes [32]. The situation at the potato *R1 *locus described here remarkably resembles this finding. The R1-contig is part of a genome fragment of unknown size introgressed from *S. demissum *into *S. tuberosum*, whereas the r1-contig originated either from *S. tuberosum *or *S. spegazzinii*, another closely related tuber bearing *Solanum *species. P40, the parental donor of the *r1 *allele, was an inter-specific hybrid between *S. tuberosum *and *S. spegazzinii *[33]. The structural differences between the homologous chromosomes could interfere with chromosome pairing and crossing-over during meiosis, explaining the low frequency of recombination observed in this region [22]. Similarly, in regions on tomato chromosome 6 and 11, where the resistance loci *Mi *and *Tm2a*, respectively, have been introgressed from the wild species *S. peruvianum*, a high degree of recombination suppression was observed [32,34].

In the R1 contig, the inversion seems to have separated the *R1 *resistance gene from a tandem array of R1 homologs (proteins 22, 23 and 24). We attempted to date the inversion relative to the duplications of tandem resistance genes by phylogenetic analysis of the protein sequences, but the results were inconclusive (data not shown). Proteins 23, 24, 44 and 45 clustered together. Proteins 22 and 22/1 also clustered together, and even though gene 22 on the R1 contig is truncated at the N-terminus relative to 22/1 and the other R1 homologous proteins, we assume these to be an orthologous pair. For the other *R1 *homologues, allelic relationships are not clear, and they may have arisen through duplication after the divergence of R1 and r1.

Kuang et al [25] analyzed the same genomic region on potato chromosome V between three homeologous chromosomes of the allo-hexaploid potato species *S. demissum*. Alignment of the sequenced *S. demissum *BACs with the R1- and r1-contig identified haplotypes A and B/C as most similar but not identical to the R1- and r1-contig, respectively. Similarity of individual homologous protein pairs between B and r1 or C and r1 ranges between 90 and 99% identity on the amino acid level, with most, but not all proteins slightly more similar between C and r1 than between B and r1. On the other hand, the B haplotype is structurally more similar to r1, as C shows no tandem repeated *R1 *homologous genes but only one single copy.

**Figure 3:**
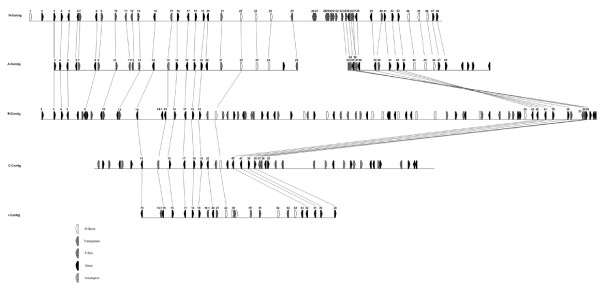
Comparison between gene loci of the R1- and r1 contig as well as of the A, B and C haplotype of *S. demissum*. White polygons symbolize R-genes. Striped polygons indicate pseudogenes. Chequered polygons represent F-box genes. Gray polygons stand for transposons and all other genes are shown as black polygons. Orthologous genes are connected by black lines.

Remarkable is the discovery of highly conserved and seemingly rapidly evolving genome regions in close vicinity. The distinguishing feature, aside from the observed breakdown of nucleotide sequence similarity in non-coding regions and the lack of gene-by-gene co-linearity, is the presence of tandem repeated genes, namely *R1 *homologs and F-box containing genes. Such tandem arrays have been found in other hypervariable genome regions [35] and may have lead to a greatly enhanced rate of evolution due to relaxed selection pressure on duplicated genes. Neighboring unique sequences are, in contrast, highly conserved. In the variable region, the R1 and r1 contigs and the three *S. demissum *haplotypes show striking structural variation, ranging from lack of the variable region in *S. demissum *haplotype C to the expanded set of F-box proteins found in the R1 contig. The latter are missing from r1 and B. Instead, in the B haplotype the *R1 *homologous gene tandem array is more expanded (figure 3).

### Encoded proteins

We found a gene density of one gene every 9 kb, which is similar to previous findings in *S. demissum *(7.6 kb, [24]) and tomato (8 kb, [36,37]), but lower than *A. thaliana *(5 kb, [38]) and rice (6 kbp, [39]), and higher than in barley, where only three genes were found in a stretch of 60 kbp genomic DNA [40]. The overall GC content was 37% and 39.5% within the putative gene coding regions. These values are comparable to tomato (37% overall GC content and 42% in coding regions, [36]) and *A. thaliana *(36% overall GC content and 44% in coding regions, [38]) but lower than in rice (44% overall GC content and 54% in coding regions, [39]) and maize (47% overall GC content and 55% in coding regions.

### The R1 gene family and disease resistance QTL

Annotation of the *R1 *gene family in *S. demissum *haplotype A [25] and in the R1-contig was comparable but not identical. In the R1-contig, six putative full-length *R1 *homologous genes and no partial homolog were annotated besides the *R1 *resistance gene itself. The six members of the *R1 *gene family were organized in two clusters of three genes each (proteins 22, 23, 24 and 44 = *R1*, 45, 46). In the corresponding region of *S. demissum *haplotype B, four putative full length *R1 *homologous genes and six partial homologues were identified. In *S. demissum *haplotype C, one complete *R1 *homologue was found [24], whereas three complete members of the *R1 *gene family (proteins 22/1, 52 and 54) were annotated in contig r1(Figures 1 and 3). Allelic relationships between the *R1 *homologues could not be deduced with certainty, but proteins 22 and 22/1 might be allelic based on collinear positions in the R1- and r1-contig, whereas proteins 44 and 45 might be allelic to 54 and 52 respectively as they are collinear under the assumption that they are part of the proposed genomic inversion.

Most of the molecular characterized plant *R *genes are members of tightly linked gene families [41]. The *R1 *gene family is no exception in this respect. Allelic variants of the nine identified members of the *R1 *family or additional paralogous members that are not present in genotype P6/210 are non-exclusive candidates for the quantitative resistance traits in the resistance hot spot on potato chromosome V. At this point, we only know that some of the *R1 *homologues likely have functions other than the *R1 *resistance gene. This is based on the observation that the *R1 *homologue encoded by ORF 45 was not capable to complement the *R1 *race specific resistance phenotype (A. B. unpublished results).

Genes sequence-related to retrotransposons, ribosomal genes, RNA dependent RNA polymerase and pseudogenes are ranked low for being functional candidates for the quantitative traits in this region of the potato genome. The remaining 30 putative genes, including hypothetical genes and genes with unknown function, are all positional candidates for the QTL. Of particular interest as new candidates for quantitative resistance loci, besides the members of the *R1 *gene family, are the members of the F-box domain family. F-box proteins are involved in various signaling pathways in *A. thaliana*, and recently, F-box proteins are suggested to function as receptors for various plant hormones [42]. Furthermore, an F-box domain was identified in the SGT1 protein that was shown to play a role as co-chaperon in the stabilization of R-proteins [43,44]. With the annotation of the sequenced region, the list of positional candidate genes for the QTL is certainly not complete, as the sequence covers only part of the *GP21–GP179 *interval. To ultimately validate the role of any candidate gene for a QTL, complementation analysis with allelic variants is required. Unless high-throughput methods for complementation analysis become available, strategies to reduce the number of candidate genes to be considered for complementation analysis are necessary. For example, we perform functional testing by expression studies and by down-regulation of candidate gene expression by antisense or RNAi approaches [45]. The model plant *A. thaliana *may also be used to study the function of genes that are most closely sequence-related to potato positional candidate genes. Unfortunately, this approach may not be applicable to the F-box family, as this is also a highly expanded gene family in *A. thaliana *with diverse cellular roles.

### Synteny with *A. thaliana*

We identified at least five microsyntenic relationships between the R1 contig and *A. thaliana*. These cover varying stretches of the genome, ranging from just four consecutive genes within 7 kb of *A. thaliana *and 25 kb of potato to basically the entire R1 contig, covering 405 kb. Frequent insertion-deletion events can be detected. Similar patterns of interrupted co-linearity on the DNA sequence level were found among cereals [46] and between *A. thaliana *and rice [47]. In the highly collinear tomato genome [2,5], genomic sequences of 57 kbp and 106 kbp on chromosome 2 [48] and 7 [37], respectively, have been compared to the *A. thaliana *genomic sequence. These studies revealed syntenic blocks of comparable redundancy and size with respect to the *A. thaliana *syntenic regions. In contrast to these previous studies, the contiguous potato sequence compared was 4 to 8-times longer. This revealed that the syntenic potato genes in the R1-contig were organized in three clusters (ORF 2 to 5, ORF 17 to 19 and ORF 38 to 48) that were separated by two non-syntenic regions (ORF 6 to 16 and ORF 20 to 37). In the r1-contig, a non-syntenic region (ORF 20 to 54) separated two syntenic regions (ORF 17 to 19 and ORF 38 to 43).

The most notable of the syntenic relationships spans almost the complete R1 contig (405 kbp) and 54 kbp of *A. thaliana *chromosome 1. Seven genes are conserved in sequence, order and orientation, except for two from region E that show reverse order and orientation compared to *A. thaliana*. This could indicate that the genomic inversion occurred in the R1 lineage after the divergence of *A. thaliana *and potato, with r1 and *S. demissum *B and C haplotypes showing the ancestral orientation. In the R1 contig, a large number (18) of genes do not show synteny, whereas in the *A. thaliana *region this only applies to five genes. The discrepancy is less pronounced if the 17 tandemly duplicated genes in potato are ignored.

The non-syntenic regions correspond to the highly divergent regions between R1 and r1 and included all but one (ORF 40) transposon sequences, all F-box-containing genes and six of the ten resistance-gene-homologues. The annotation of the *A. thaliana *syntenic regions identified, besides the sequence related ORFs, some transposon sequences but only one F-box-containing gene and no resistance gene homolog. Moreover, the non-syntenic regions in the R1- and r1-contigs coincided with regions II and IV in *S. demissum*, which showed the highest divergence between the homeologous chromosome segments A, B and C [25]. This suggests that the genome of potato and related species in the sequenced region consists of a patchwork of faster and more slowly evolving segments.

The sequenced potato genomic segment covers a genetic distance of only 0.1 Centimorgan. At a hundred times larger scale, when genome-wide genetic maps of potato, sunflower, sugar beet and *Prunus *were compared to the *A. thaliana *physical map (macrosynteny), syntenic blocks from 1 to 20 Centimorgans were identified. A common fraction of the genomes of these distantly related plant species appear to have been conserved throughout the evolution of the dicots, when compared to the rest of the genome [49]. The *GP21–GP179 *interval was not part of a macrosyntenic block between potato and *A. thaliana *[4].

## Conclusion

Two contiguous sequences of 417,445 and 202,781 base pairs were assembled and annotated for a region on potato chromosome V, which contains genes controling several agronomic traits. Comparative sequence analysis revealed highly conserved collinear regions that flank regions showing high variability and tandem duplicated genes. The co-linearity between the homologous chromosomes was disrupted by non-allelic insertions of retrotransposon elements, stretches of diverged intergenic sequences, differences in gene content and gene order. The latter was mainly caused by inversion of a 70 kbp genomic fragment.

Annotation of the genomic sequence identified 48 putative open reading frames (ORF) in one contig and 22 in the other, with an average of one ORF every 9 kbp. The majority of the ORFs were members of multiple gene families. Ten ORFs were classified as resistance-gene-like, 11 as F-box-containing genes, 13 as transposable elements and three as transcription factors. Comparing potato to *Arabidopsis thaliana *annotated proteins revealed five micro-syntenic blocks of three to seven ORFs with *A. thaliana *chromosomes 1, 3 and 5, suggesting fragmented structural conservation between these distantly related plant species.

## Methods

### BAC-libraries

For contig construction, two BAC genomic libraries were used, each consisting of ca. 100 000 clones (two hundred and sixty four 384-well microtiter plates). Both libraries were generated from high molecular weight DNA of the diploid, heterozygous potato clone P6/210, a F1 hybrid of the parental clones P41 (H79.1506/1) and P40 (H80.696/4) [9]. The 'BA' library has been described [22]. The library 'BC' was constructed in the cloning vector pBeloBAC11 [50] from partially *Eco*RI digested genomic DNA. The procedures for the construction of recombinant BAC clones, clone picking and storing were as described previously [22,51]. The average insertion size of the 'BC' library was 80 kbp, corresponding to an, on average, 8-fold coverage of the potato genome.

### BAC plasmid DNA isolation

A single colony was pre-cultured in 250 μl LB medium including 12.5 mg/l tetracycline for clones from the "BA" library and 12.5 mg/l chloramphenicol for clones from the "BC" library. The pre-culture was used to inoculate 50 ml LB medium containing the corresponding antibiotic. Plasmid DNA was isolated from 50 ml overnight culture using the QIAGEN Plasmid Midi Kit (Qiagen, Hilden, Germany) according to the manufacturer's instructions.

### BAC-library screening

High-density colony filters of the libraries were prepared and screened by colony-hybridization as described [22]. The 'BC' library was also screened by the polymerase chain reaction (PCR) after isolating plasmid DNA from bacterial cells pooled at three levels: mini-pools, maxi-pools and super-pools. One thousand fifty six mini-pools were made from 96 clones each, 264 maxi-pools were prepared from 384 clones each (four mini-pools) and the 88 super-pools consisted of 1152 clones each (three maxi-pools). To identify a single positive clone, four rounds of PCR screening were performed. First, the DNA of the 88 super-pools was used as template. Second, the three maxi-pools constituting a positive super-pool were screened. Third, the four mini-pools of a positive maxi-pool were amplified and last, the 96 clones of the positive mini-pool were screened individually.

### Physical mapping

Overlapping BACs were identified and ordered in two contigs corresponding to the two homologous chromosomes of genotype P6/210 as described previously [22]. In short, BAC insertion ends were sequenced using T3 and T7 oligonucleotides as sequencing primers. The end sequences were used to detect overlaps, either based on 100% sequence identity with already sequenced BACs or by generating amplicons with identical sequences in different BACs. BAC contigs were assigned to either one or the other homologous chromosome by identifying DNA polymorphisms in insertion end sequences that were specific either for the allele inherited from parent P41 or parent P40.

### Genomic DNA Sequencing and Assembly

Whole BAC clones were sequenced by the shotgun sequencing strategy. Custom sub-libraries of the BACs were prepared by GATC-Biotech AG (Konstanz, Germany). After physical fractionation of the BAC DNA, the random sheared fragments were blunt-ended by using T4 DNA-Polymerase and then ligated into the pCR4Blunt-TOPO vector (Invitrogen, California, USA). Approximately 1300 clones containing ca. 1.5 kbp insertions and 300 to 400 clones with 4 to 5 kbp insertions were produced for each BAC. The smaller inserts were amplified by colony-PCR using as primers TO2f (5'-agcggataacaatttcacacagga-3') and TO2r (5'-gacgttgtaaaacgacggccagtg-3'). The PCR was performed in a volume of 100 μl containing 10 pmol of each primer, 0.2 mM dNTPs and 1.5 mM MgCl_2_, 0.2 Units of Taq-Polymerase and the corresponding buffer from Invitrogen, California, USA. PCR conditions were: initial denaturation at 96°C for 5 min followed by 34 cycles of denaturation at 94°C for 50 sec, annealing at 55°C for 50 sec and extension at 72°C for 3 min and a final extension for 4 min at 72°C. Plasmid DNA was purified from the clones having 5 kb inserts using the BioRobot 9600 (Qiagen, Hilden, Germany). PCR products and plasmids were sequenced using T3 and T7 primers (Amersham, Pharmacia GE Healthcare Bio-Sciences: Little Chalfont, UK). Sequencing reactions were performed by using the ABI PRISM BigDye Terminator Cycle Sequencing Ready Reaction Kit and an ABI377 automated DNA Sequencer (PE Biosystems, Foster City, California, USA). The approximately ten-fold redundant sequences were assembled using the PreGAP4 and GAP4 from Staden software package (Medical Research Council Laboratory of Molecular Biology, Cambridge, UK). The Lasergene software package (DNAstar, Madison, WI, USA) was used for sequence assemblies, comparisons and alignments. The sequences of overlapping BAC insertions were assembled after trimming any vector sequences using the SeqMan module of Lasergene and the Megamerger program, which is part of the EMBOSS package [52].

### Sequence analysis

Dotter and MUMer [54] were used to align and compare genomic sequences. Putative exons and open reading frames (ORFs) were predicted by the programs GenMark.hmm [54], FGeneSH [55] and by alignment of EST (Expressed Sequence Tags) and protein sequences using GenomeThreader [56]. Genes were annotated by combining predicted ORFs from these gene finder programs with alignments of homologous sequences in public databases using the Apollo Genome Annotation Curation Tool [57] (Additional files 1 and 2). All genes were also manually annotated for putative function. Functional descriptions of homologous genes in the SWISSPROT database [58] were compared with homologous protein domains and patterns in the InterPro database [59]. Homologous genes in the SWISSPROT database were identified using BlastP [60] and homologous protein domains and patterns were identified by InterProScan [61]. Inparanoid [62] and BlastX [60] were used for the identification of similar genes in *A. thaliana*. The deduced amino acid sequences of the annotated ORFs were compared to *A. thaliana *annotated proteins from The A. thaliana Information Resource (TAIR) Release 6 [64]. The threshold criterion for accepting sequence similarity as significant was an E-value < 10^-10 ^for BLASTP searches. Polyproteins and transposable elements were excluded from the comparison because of their limited information value. For the same reason, ORFs with more than 30 hits in the *A. thaliana *genome were also excluded [4]. A two-dimensional array was generated with the potato physical map of 420,000 bp in one dimension and the A. thaliana physical map of 121 mb in the other [4]. Hits of the putative potato proteins with *A. thaliana *annotated proteins were positioned in the array according to their base pair coordinates on the local potato and genome wide *A. thaliana *physical maps. Syntenic blocks were identified based on the criterion that at least three different ORFs within the potato contigs found hits within an *A. thaliana *genome fragment of similar size.

## List of abbreviations

QTL, quantitative trait loci; QRL, quantitative resistance loci; ORF, open reading frame; RGL, resistance-gene-like; EST, Expressed Sequence Tags; BAC, bacterial artificial chromosome; PCR, polymerase chain reaction; cM, Centimorgan; kbp, kilo base pairs; mb, mega base;

## Authors' contributions

AB designed and conducted the experiments for constructing the physical map and BAC sequencing, did sequence annotation, synteny analysis and drafted the manuscript. HS, AJ and RB provided bioinformatics support (sequence analysis, sequence comparisons and ORF annotation). PV and BW contributed to BAC sequencing. HI, JP and KM constructed the BC-BAC library. CG and HS contributed substantially to manuscript preparation and editing. CG designed and oversaw the project.

## Supplementary Material

Additional File 5 Table S1: Genomic sequence annotation of the R1- and r1-contig Click here for file

Additional File 6 Table S2: Putative orthologs from R1-, r1-contig and haplotypes A, B and C of *S. demissum *and the corresponding accession numbers are shown. Putative pseudogenes are indicated by "PS". The identities between transposons (TP) are not shown. Click here for file

Additional File 3 Figure S3: Dot-Plot comparison computed by MUMmer of the DNA sequence of the R1-contig and individual BAC sequences of *S. demissum*. One column represents one BAC sequence of *S. demissum*. The first three columns (from left to right) are from haplotype A, the next seven from haplotype B and the last two columns from haplotype C. The figure shows high similarity and co-linearity between the R1-contig and haplotype A of *S. demissum*. The first BAC (PGEC446J10, AC149228) is shown in reverse orientation. The region between 270 and 320 kb on the R1-contig is not covered by the non-overlapping BACs PGEC668E02 (AC144791) and PGEC472P22 (AC151815), so we can estimate the gap between these in haplotype A to be around 50 kb in size, covered by the unsequenced BAC PGEC597C19 according to Kuang et al. Only local and limited similarity and co-linearity is observed between the R1-contig and BACs from the B and C haplotypes. The colour reflects percent sequence similarity, with red denoting close to 100% similarity. Click here for file

Additional File 4 Figure S4: Dot-Plot comparison computed by MUMmer of the DNA sequence of the r1-contig and BAC sequences of *S. demissum*. One column represents one BAC sequence of *S. demissum*. The first three columns (from left to right) are BAC sequences from haplotype A, the next seven from haplotype B and the last two columns from haplotype C. The figure shows two regions of high similarity and co-linearity between the r1-contig and haplotype B and C of *S. demissum*, with similar but weaker similarity to haplotype A sequences. The first comprises the region between the beginning and 60 kb of the r1-contig and the second runs from 160 kb till the end of the r1-contig. The middle region shows some local similarity that is interrupted by non-conserved sequence. Most of the more significant similarities in this area reflect tandemly duplicated R-genes. The BAC sequences from haplotype C do not contain this region, indicating a deletion in haplotype C as found by Huang et al. An inverted repeat of a RNA-dependent RNA polymerase leads to an X-shaped structure in the plot of the first 40 kb of the r1-contig against the *S. demissum *sequences, similar to the findings in Figure 2 when comparing to the R1-contig (region B). The colour reflects percent sequence similarity, with red denoting close to 100% similarity. Click here for file

Additional File 1 Figure S1: Gene annotation of R1-contig using APOLLO Click here for file

Additional File 2 Figure S2: Gene annotation of r1-contig using APOLLO Click here for file

## References

[B1] Gebhardt C Lörz H, Wenzel G Potato Genetics: Molecular Maps and More in Biotechnology in Agriculture and Forestry Molecular Marker Systems 2004 55 Springer-Verlag, Berlin, Heidelberg 215 227

[B2] Bonierable M Plaisted RL Tanksley SD RFLP map based on a common set of clones reveal modes of chromosomal evolution in potato and tomato Genetics 1988 120 1095 1103 17246486 10.1093/genetics/120.4.1095 PMC1203572

[B3] Gebhardt C Ritter E Debener T Schachtschabel U Walkemeier B Uhrig U Salamini F RFLP analysis and linkage mapping in *Solanum tuberosum* Theor Appl Genet 1989 78 65 75 10.1007/BF00299755 24227032

[B4] Gebhardt C Walkemeier B Henselewski H Barakat A Delseny M Stüber K Comparative mapping between potato (*Solanum tuberosum*) and *A. thaliana *reveals structurally conserved domains and ancient duplications in the potato genome The Plant J 2003 34 529 541 10.1046/j.1365-313X.2003.01747.x 12753591

[B5] Tanksley SD Ganal MW Prince JP de Vicente MC Bonierbale MW Broun P Fulton TM Giovannoni JJ Grandillo S Martin GB Messeguer R Miller JC Miller L Paterson AH Pineda O Roeder MS Wing RA Wu W Young ND High density molecular linkage maps of the tomato and potato genomes Genetics 1992 132 1141 1160 1360934 10.1093/genetics/132.4.1141 PMC1205235

[B6] Gebhardt C Valkonen JPT Organization of genes controlling disease resistance in the potato genome Annu Rev Phytopathol 2001 39 79 102 10.1146/annurev.phyto.39.1.79 11701860

[B7] Meksem K Leister D Peleman J Zabeau M Salamini F Gebhardt C A high-resolution map of the vicinity of the *R1 *locus on chromosome V of potato based on RFLP and AFLP markers Mol Gen Genet 1995 249 74 81 10.1007/BF00290238 8552036

[B8] De Jong W Forsyth A Leister D Gebhardt C Baulcombe DC A Potato hypersensitive resistance gene against potato virus X maps to a resistance gene cluster on chromosome V Theor Appl Genet 1997 5 153 162

[B9] Ritter E Debener T Barone A Salamini F Gebhardt C RFLP mapping on potato chromosomes of two genes controlling extreme resistance to potato virus X (PVX) Mol Gen Genet 1991 227 81 85 10.1007/BF00260710 1675423

[B10] Leonards-Schippers C Gieffers W Gebhardt C Salamini F The *R1 *gene conferring race-specific resistance to *Phytophtora infestans *in potato is located on potato chromosome V Mol Gen Genet 1992 233 278 283 10.1007/BF00587589 1351246

[B11] Leonards-Schippers C Gieffers W Schäfer-Pregl R Ritter E Knapp SJ Salamini F Gebhardt C Quantitative resistance to *Phytophthora infestans *in potato: a case study for QTL mapping in an allogamous plant species Genetics 1994 137 67 77 7914505 10.1093/genetics/137.1.67 PMC1205955

[B12] Oberhagemann P Chatot-Balandras C Bonnel E Schäfer-Pregl R Wegener D Palomino C Salamini F Gebhardt C A genetic analysis of quantitative resistance to late blight in potato: Towards marker assisted selection Mol Breed 1999 5 399 415 10.1023/A:1009623212180

[B13] Collins A Milbourne D Ramsay L Meyer R Chatot-Balandras C Oberhagemann P De Jong W Gebhardt C Bonnel E Waugh R QTL for field resistance to late blight in potato are strongly correlated with earliness and vigour Mol Breed 1999 5 387 398 10.1023/A:1009601427062

[B14] Visker MHPW Keizer LCP Van Eck HJ Jacobsen ELT Colon LT Struik PC Can the QTL for late blight resistance on potato chromosome 5 be attributed to foliage maturity type? Theor Appl Genet 2003 106 317 325 12582858 10.1007/s00122-002-1021-2

[B15] Kreike CM De Koning JRA Vinke JH Van Ooijen JW Stiekema WJ Quantitatively-inherited resistance to *Globodera pallida *is dominated by one major locus in *Solanum spegazzinii* Theor Appl Genet 1994 88 764 769 10.1007/BF01253983 24186175

[B16] Rouppe van der Voort J Wolters P Folkertsma R Hutten R van Zandvoort P Vinke H Kanyuka K Bendahmane A Jacobsen E Janssen R Bakker J Mapping of the cyst nematode resistance locus *Gpa2 *in potato using a strategy based on co-migrating AFLP markers Theor Appl Genet 1997 95 874 880 10.1007/s001220050638

[B17] Rouppe van der Voort J van der Vossen E Bakker E Overmars H van Zandvoort P Hutten R Klein-Lankhorst R Bakker J Two additive QTLs conferring broad-spectrum resistance in potato to *Globodera pallida *are localized on resistance gene clusters Theor Appl Genet 2000 101 1122 30 10.1007/s001220051588

[B18] Sattarzadeh A Achenbach U Lübeck J Strahwald J Tacke E Hofferbert HR Rotstein T Gebhardt C Single nucleotide polymorphism (SNP) genotyping as basis for developing a PCR-based marker highly diagnostic for potato varieties with high resistance to *Globodera pallida *pathotype Pa2/3 Mol Breed 2006 4 301 312 10.1007/s11032-006-9026-1

[B19] Schäfer-Pregl R Ritter E Concilio L Hesselbach J Lovatti L Walkemeier B Thelen H Salamini F Gebhardt C Analysis of quantitative trait loci (QTL) and quantitative trait alleles (QTA) for potato tuber yield and starch content Theor Appl Genet 1998 97 834 846 10.1007/s001220050963

[B20] Menendez CM Ritter E Schäfer-Pregl R Walkemeier B Kalde A Salamini F Gebhardt C Cold-sweetening in diploid potato. Mapping QTL and candidate genes Genetics 2002 162 1423 1434 12454085 10.1093/genetics/162.3.1423 PMC1462350

[B21] Bendahmane A Querci M Kanyuka K Baulcombe DC Agrobacterium transient expression system as a tool for the isolation of disease resistance genes: application to the *Rx2 *locus in potato Plant J 2000 21 73 81 10.1046/j.1365-313x.2000.00654.x 10652152

[B22] Ballvora A Ercolano MR Weiss J Meksem K Bormann CA Oberhagemann P Salamini F Gebhardt C The *R1 *gene for potato resistance to late blight (*Phytophthora infestans*) belongs to the leucine zipper/NBS/LRR class of plant resistance genes The Plant J 2002 30 361 371 10.1046/j.1365-313X.2001.01292.x 12000683

[B23] Chisholm ST Coaker G Day B Staskawicz BJ Host-Microbe Interactions: Shaping the Evolution of the Plant Immune Response Cell 2006 124 803 814 10.1016/j.cell.2006.02.008 16497589

[B24] Ross H Potato breeding. Problems and perspectives Adv Plant Breed 1986 Supplement 13 Paul Parey Verlag, Berlin and Heidelberg

[B25] Kuang H Wei F Marano MR Wirtz U Wang X Liu J Shum WP Zaborsky J Tallon LJ Rensink W Lobst S Zhang P Tornqvist CE Tek A Bamberg J Helgeson J Fry W You F Luo MC Jiang J Robin Buell C Baker B The *R1 *resistance gene cluster contains three groups of independently evolving, type I *R1 *homologues and shows substantial structural variation among haplotypes of *Solanum demissum* The Plant J 2005 44 37 51 10.1111/j.1365-313X.2005.02506.x 16167894

[B26] Pryor T Ellis J The genetic complexity of fungal resistance genes in plants Adv Plant Pathol 1993 10 281 305

[B27] Gebhardt C Ballvora A Walkemeier B Oberhagemann P Schüler K Assessing genetic potential in germ plasm collections of crop plants by marker-trait association: a case study for potatoes with quantitative variation of resistance to late blight and maturity type Mol Breed 2004 13 93 102 10.1023/B:MOLB.0000012878.89855.df

[B28] Salvi S Tuberosa R To clone or not to clone plant QTLs: present and future challenges Trends Plant Sci 2005 10 297 304 10.1016/j.tplants.2005.04.008 15949764

[B29] Stein L Genome annotation: From sequence to biology Nature Reviews Genetics 2001 2 493 503 10.1038/35080529 11433356

[B30] Fu H Dooner HK Intraspecific violation of genetic colinearity and its implications in maize Proc Natl Acad Sci 2002 99 9573 9578 12060715 10.1073/pnas.132259199 PMC123182

[B31] Brunner S Fengler K Morgante M Tingey S Rafalski A Evolution of DANN Sequence Nonhomologies among Maize Inbreds Plant Cell 2005 17 343 360 10.1105/tpc.104.025627 15659640 PMC548811

[B32] Seah S Yaghoobi J Rossi M Gleason CA Williamson VM The nematode gene, Mi-1, is associated with an inverted chromosomal segment in susceptible compared to resistant tomato Theor Appl Genet 2004 108 1635 1642 10.1007/s00122-004-1594-z 14963654

[B33] Barone A Ritter E Schachtschabel U Debener T Salamini F Gebhardt C Localization by restriction fragment length polymorphism mapping in potato of a major dominant gene conferring resistance to the potato cyst nematode *Globodera rostochiensis* Mol Gen Genet 1990 224 177 182 10.1007/BF00271550 1980523

[B34] Ganal MW Tanksley SD Recombination around *Tm2a *and *Mi *resistance genes in different crosses of *Lycopersicom peruvianum* Theor Appl Genet 1996 92 101 108 10.1007/BF00222958 24166123

[B35] Michelmore RW Meyers BC Clusters of resistance genes in plants evolve by divergent selection and a birth-and-death process Genome Res 1998 8 9828 9832 10.1101/gr.8.11.1113 9847076

[B36] Mao l Begum D Goff SA Wing RA Sequence and Analysis of the Tomato *JOINTLESS *Locus Plant Physiol 2001 126 3 1331 1340 10.1104/pp.126.3.1331 11457984 PMC116490

[B37] Rossberg M Theres K Acarkan A Herrero R Schmitt T Schumacher K Schmitz G Schmidt R Comparative sequence analysis reveals extensive microcolinearity in the *Lateral Suppressor *regions of the tomato, *A. thaliana*, and *Capsella *genomes The Plant Cell 2001 13 979 988 10.2307/3871354 11283350 PMC135537

[B38] The A. thaliana Genome Initiative Analysis of the genome sequence of the flowering plant *A. thaliana* Nature 2000 408 796 815 10.1038/35048692 11130711

[B39] The Rice Chromosome 3 Sequencing Consortium Sequence, annotation, and analysis of synteny between rice chromosome 3 and diverged species Genome 2006 15 1284 1291 10.1101/gr.3869505 PMC1199543 16109971

[B40] Panstruga R Büschges R Piffanelli P Schulze-Lefert P A contigous 60 kb genomic stretch from barley reveals molecular evidence for gene islands in a monocot genome Nuc Acid Res 1998 26 1056 1062 10.1093/nar/26.4.1056 PMC147355 9461468

[B41] Hulbert SH Webb CA Smith SM Sun Q Resistance gene complexes: evolution and utilization Annu Rev Phytopathol 2001 39 285 312 10.1146/annurev.phyto.39.1.285 11701867

[B42] Kepinski S Leyser O The A. thaliana F-box protein TIR1 is an auxin receptor Nature 2005 435 446 451 10.1038/nature03542 15917798

[B43] Shirazu K Schulze-Lefert P Regulators of cell death in disease resistance Plant Mol Biol 2000 44 371 385 10.1023/A:1026552827716 11199395

[B44] Schulze-Lefert P Plant Immunity: The origami of receptor activation Curr Biol 2004 14 R22 R24 14711430

[B45] Waterhause PM Helliwell CA Exploring plant genomes by RNA-induced gene silencing Nat Rev Genet 2003 4 29 38 10.1038/nrg982 12509751

[B46] Ware D Stein L Comparison of genes among cereals Curr Op Plant Biol 2003 6 121 127 10.1016/S1369-5266(03)00012-8 12667867

[B47] Bennetzen JL Ma J The genetic colinearity of rice and other cereals on the basis of genomic sequence analysis Curr Op Plant Biol 2003 6 128 133 10.1016/S1369-5266(03)00015-3 12667868

[B48] Ku H.-M Vision T Liu J Tanksley SD Comparing sequenced segments of the tomato and *A. thaliana *genomes: Large-scale duplication followed by selective gene loss creates a network of synteny Proc Natl Acad Sci 2000 97 9121 9126 10.1073/pnas.160271297 10908680 PMC16832

[B49] Dominguez I Graziano E Gebhardt C Barakat A Berry S Arús P Delseny M Barnes S Plant genome acheology: evidence for conserved ancestral chromosome segments in dicotyledonous plant species Plant Biotech J 2003 1 91 99 10.1046/j.1467-7652.2003.00009.x 17147746

[B50] Kim UJ Birren BW Slepak T Mancino V Boyson C Kang HL Simon MI Shizuya H Construction and characterization of a human bacterial artificial chromosome library Genomics 1996 34 213 218 10.1006/geno.1996.0268 8661051

[B51] Meksem K Zobrist K Ruben E Hyten D Quanzhou T Zhang H-B Lightfoot DA Two large-insert soybean genomic libraries constructed in a binary vector: applications in chromosome walking and genome wide physical mapping Theor Appl Genet 2000 101 747 755 10.1007/s001220051540

[B52] Rice P Longden I Bleasby A EMBOSS: the European Molecular Biology Open Software Suite Trends Genet 2000 16 6 276 7 10.1016/S0168-9525(00)02024-2 10827456

[B53] Sonnhammer EL Durbin R A dot-matrix program with dynamic threshold control suited for genomic DNA and protein sequence analysis Gene 1995 167 GC1 GC10 10.1016/0378-1119(95)00714-8 8566757

[B54] Lukashin AV Borodovsky M GeneMark.hmm: new solutions for gene finding Nuc Acids Res 1998 26 1107 1115 10.1093/nar/26.4.1107 PMC147337 9461475

[B55] Salamov AA Solovyev VV Ab initio Gene Finding in *Drosophila *Genomic DNA Genome Res 2000 10 516 522 10.1101/gr.10.4.516 10779491 PMC310882

[B56] Gremme G Brendel V Sparks ME Kurtz S Engineering a software tool for gene structure prediction in higher organisms Information and Software Technology 2005 47 965 978 10.1016/j.infsof.2005.09.005

[B57] Lewis SE Searle SMJ Harris N Gibson M Iyer V Ricter J Wiel C Bayraktaroglu L Birney E Crosby MA Kaminker JS Matthews B Prochnik SE Smith CD Tupy JL Rubin GM Misra S Mungall CJ Clamp ME Apollo: a sequence annotation editor Genome Biol 2002 3 research0082 10.1186/gb-2002-3-12-research0082 12537571 PMC151184

[B58] Bairoch A Apweiler R The SWISS-PROT protein sequence database and its supplement TrEMBL in 2000 Nuc Acids Res 2000 28 45 48 10.1093/nar/28.1.45 PMC102476 10592178

[B59] Apweiler R Attwood TK Bairoch A Bateman A Birney E Biswas M Bucher P Cerutti L Corpet F Croning MDR Durbin R Falquet L Fleishmann W Gouzy J Hermjakob H Hulo N Jonassen L Kahn D Kanapin A Karavidopoulou Y Lopez R Marx B Mulder NJ Oinn TM Pagni M Servant P Sigrist CJA Zdobnov EM The InterPro database, an integrated documentation resource for protein families, domains and functional sites Bioinformatics 2000 16 1145 1150 10.1093/bioinformatics/16.12.1145 11159333

[B60] Altschul F Madden TL Schaffer AA Zhang J Zhang Z Miller W Lipman DJ Gapped BLAST and PSI-BLAST: a new generation of protein database search programs Nuc Acids Res 1997 25 3389 3402 10.1093/nar/25.17.3389 PMC146917 9254694

[B61] Zdobnov EM Apweiler R InterProScan – an integration platform for the signature-recognition methods in InterPro Bioinformatics 2001 17 847 848 10.1093/bioinformatics/17.9.847 11590104

[B62] Remm M Storm CEV Sonnhammer ELL Automatic Clustering of Orthologs and In-paralogs from Pairwise Species Comparisons JMB 2001 14 1041 1052 10.1006/jmbi.2000.5197 11743721

[B63] GABI Primary Database https://gabi.rzpd.de/projects/Pomamo/

[B64] The Arabidopsis Information Resource http://www.arabidopsis.org

