# Supplementary material for: Comparative sequence analysis of Solanum and Arabidopsis in a hot spot for pathogen resistance on potato chromosome V reveals a patchwork of conserved and rapidly evolving genome segments
Source: BMC Genomics. 2007 May 2;8:112. doi: 10.1186/1471-2164-8-112 (PMC3225836; doi:10.1186/1471-2164-8-112)
Supplement: Additional File 5 — Table S1: Genomic sequence annotation of the R1- and r1-contig [file 1471-2164-8-112-S5.doc]

Table S1. The annotated ORFs from the contigs numbered as defined by APOLLO and as shown schematically in Figure 2. Manual functional annotation is done based on BLAST hits and/or domain prediction results. Sign “ - “ indicates that no hits or only hits with e-values higher than 1^-10^ were retrieved. The accession numbers and the corresponding e-values are the best hits when predicted ORF protein sequence is blasted against SWISSPROT database. Conserved domains are defined using InterPro and the accession numbers with the corresponding description are shown.

| ORF Number | Manual functional annotation | Best hit in SwissProt | | | Domain Prediction(InterPro) | |
| --- | --- | --- | --- | --- | --- | --- |
|  |  | Accession | Description | e-value | Accession | Description |
| 1 | Fragment of disease resistance protein | Q9M667 | Disease resistance protein RPP13 (Resistance to Peronospora parasitica protein 13) | 3e-08 | IPR002182  SSF52540 | NB-ARC domain  P-loop containing nucleoside triphosphate hydrolases |
| 2 | ZF-HD homeobox protein | Q9SB61 | ZF-HD homeobox protein At4g24660 (AtHB-22) | 1e-43 | IPR006455  IPR006456  IPR009057 | Homeobox domain, ZF-HD class  ZF-HD homeobox protein Cys/His-rich dimerisation region  Homeodomain-like |
| 3 | Protein of unknown function | - |  | - | IPR008195  IPR012870 | Ribosomal protein L34e  Protein of unknown function DUF1666 |
| 4 | Ribosomal protein L34e | P41098 | 60S ribosomal protein L34 | 3e-60 | IPR008195 | Ribosomal protein L34e |
| 5 | No apical meristem (NAM) -like | Q39013 | NAC domain-containing protein 2 (ANAC002) | 2e-39 | IPR003441    SSF54373 | No apical meristem (NAM) protein  FAD-linked reductases, C-terminal domain |
| 6 | Hypothetical protein | Q02817 | - | - | - | - |
| 7 | Retrotransposon | P04146 | Copia protein (Gag-int-pol protein) [Contains: Copia VLP protein; | 2e-68 | IPR001584  IPR012337  IPR013103  PTHR11439  SSF56672 | Integrase, catalytic region  Polynucleotidyl transferase, Ribonuclease H fold  Reverse transcriptase, RNA-dependent DNA polymerase  GAG-POL-RELATED RETROTRANSPOSON  DNA/RNA polymerases |
| 8 | Hypothetical protein | P68344 | - | - | - | - |
| 9 | Retrotransposon | P04146 | Copia protein (Gag-int-pol protein) [Contains:Copia VLP protein; Copia protease (EC 3.4.23.-)] | 9e-22 | IPR013103  PTHR11439 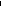  SSF56672 | Reverse transcriptase, RNA-dependent DNA polymerase  GAG-POL-RELATED RETROTRANSPOSON  DNA/RNA polymerases |
| 10 | F-box protein | - | - | - | IPR001810  IPR011043 | Cyclin-like F-box  Galactose oxidase, central |
| 11 | Pseudogene | P03200 | - | - | - | - |
| 12 | Pseudogene | - | - | - | IPR002156  IPR012337  PTHR19446  PTHR19446:SF34 | Ribonuclease H  Polynucleotidyl transferase, Ribonuclease H fold  Polynucleotidyl transferase, Ribonuclease H fold  REVERSE TRANSCRIPTASES |
| 13 | F-box protein | - | - | - | IPR001810 | Cyclin-like F-box |
| 14 | RNA dependent RNA polymerase | O14227 | RNA-dependent RNA polymerase homolog 1 (Protein rdp1) | 2e-93 | IPR007855  IPR012677  PTHR23079  SSF54928 | RNA dependent RNA polymerase  Nucleotide-binding, alpha-beta plait  RNA-DEPENDENT RNA POLYMERASE  RNA-DEPENDENT RNA POLYMERASE |
| 14/1 | Transposon fragment | - | - | - | - | - |
| 15 | Hypothetical protein | - | - | - | - | - |
| 16 | RNA dependent RNA polymerase | O14227 | RNA-dependent RNA polymerase homolog 1 (Protein rdp1) | 1e-71 (R1)  1e-93  (r1) | IPR007855  IPR012677  PTHR23079  SSF54928 | RNA dependent RNA polymerase  Nucleotide-binding, alpha-beta plait  RNA-DEPENDENT RNA POLYMERASE  RNA-DEPENDENT RNA POLYMERASE |
| 17 | CAAX amino terminal protease | - | - | - | IPR003675 | Abortive infection protein |
| 18 | Methyltransferase | - | - | - | IPR001601  IPR004159  SSF53335 | Generic methyltransferase  Protein of unknown function DUF248, methyltransferase putative  S-adenosyl-L-methionine-dependent methyltransferases |
| 19 | Phytochrome kinase substrate | Q9SWI1 | Phytochrome kinase substrate 1 | 7e-39  (R1)  5e-41  (r1) | - | - |
| 19/1 | Retrotransposon | P10978 | Retrovirus-related Pol polyprotein from transposon TNT 1-94  [Contains: Protease ; Reverse transcriptase ; Endonuclease] | e-139 | IPR001584  IPR001878  IPR005162  IPR012337  IPR013103  PTHR11439  SSF53335  SSF56672  SSF57756 | Integrase, catalytic region  Zinc finger, CCHC-type  Retrotransposon gag protein  Polynucleotidyl transferase, Ribonuclease H fold  Reverse transcriptase, RNA-dependent DNA polymerase  GAG-POL-RELATED RETROTRANSPOSON  S-adenosyl-L-methionine-dependent methyltransferases  GAG-POL-RELATED RETROTRANSPOSON  GAG-POL-RELATED RETROTRANSPOSON |
| 20 | AAA ATPase | Q9CZP5 | Mitochondrial chaperone BCS1 (BCS1-like protein) | 9e-22  (R1 and r1) | IPR003593  IPR003959  IPR003960  PTHR23070  PTHR23070:SF1  SSF52540 | AAA ATPase  AAA ATPase, central region  AAA-protein subdomain  AAA ATPase, central region  MITOCHONDRIAL CHAPERONE BCS1  P-loop containing nucleoside triphosphate hydrolases |
| 21 | Retrotransposon | P10978 | Retrovirus-related Pol polyprotein from transposon TNT 1-94 [Contains: Protease ; Reverse transcriptase ; Endonuclease] | e-122 (R1)  e-140 (r1) | IPR000871  IPR001584  IPR005162  IPR012337  IPR013103  PTHR11439  SSF56672 | Beta-lactamase, class A  Integrase, catalytic region  Retrotransposon gag protein  Polynucleotidyl transferase, Ribonuclease H fold  Reverse transcriptase, RNA-dependent DNA polymerase  GAG-POL-RELATED RETROTRANSPOSON  DNA/RNA polymerases |
| 21/1 | Disease resistent protein | Q8W1E0 | Late blight resistance protein R1-A (Protein R1) | 0 | IPR000767  IPR002182  IPR006121  PTHR22814  SSF46785  SSF52047  SSF52540 | Disease resistance protein  NB-ARC  Heavy metal transport/detoxification protein  COPPER TRANSPORT PROTEIN ATOX1-RELATED  "Winged helix" DNA-binding domain  RNI-like  P-loop containing nucleoside triphosphate hydrolases |
| 22 | Disease resistance protein | Q8W1E0 | Late blight resistance protein R1-A (Protein R1) | 0 | IPR000767  IPR002182 | Disease resistance protein  NB-ARC |
| 23 | Disease resistance protein | Q8W1E0 | Late blight resistance protein R1-A (Protein R1) | 0 | IPR000767  IPR002182  SSF46785  SSF52058  SSF52540 | Disease resistance protein  NB-ARC  "Winged helix" DNA-binding domain  L domain-like  P-loop containing nucleoside triphosphate hydrolases |
| 24 | Disease resistance protein | Q8W1E0 | Late blight resistance protein R1-A (Protein R1) | 0 | IPR000767  IPR002182  PTHR13779  SSF46785  SSF52058  SSF52540 | Disease resistance protein  NB-ARC  HOLLIDAY JUNCTION DNA HELICASE RUVB-RELATED  "Winged helix" DNA-binding domain  L domain-like  P-loop containing nucleoside triphosphate hydrolases |
| 25 | Retrotransposon | - | - | - | IPR005162  PTHR10178 | Retrotransposon gag protein  GAG/POL/ENV POLYPROTEIN |
| 26 | Hypothetical protein | - | - | - | - | - |
| 27 | Pseudogene (F-box protein-like) | - | - | - | IPR001810  IPR006527  IPR011043  SSF81383 | Cyclin-like F-box  F-box associated type 1  Galactose oxidase, central  F-box domain |
| 28 | MuDR transposase | - | - | - | IPR001878  IPR004332  IPR006564  IPR007527  SSF57756 | Zinc finger, CCHC-type  Plant MuDR transposase  Zinc finger, PMZ-type  Zinc finger, SWIM-type  Retrovirus zinc finger-like domains |
| 29 | F-box protein | - | - | - | IPR001810  IPR006527  IPR011043  SSF81383 | Cyclin-like F-box  F-box associated type 1  Galactose oxidase, central  F-box domain |
| 30 | F-box protein | - | - | - | IPR001810  IPR006527  IPR011043  SSF81383 | Cyclin-like F-box  F-box associated type 1  Galactose oxidase, central  F-box domain |
| 31 | Ataxin-2-domain containing protein | - | - | - | IPR009818 | Ataxin-2, C-terminal |
| 32 | F-box protein | - | - | - | IPR001810  IPR006527  SSF81383 | Cyclin-like F-box  F-box associated type 1  F-box domain |
| 33 | F-box protein | - | - | - | IPR001810  IPR006527  IPR011043  SSF81383 | Cyclin-like F-box  F-box associated type 1  Galactose oxidase, central  F-box domain |
| 34 | F-box associated domain containing protein | - | - | - | IPR006527 | F-box associated type 1 |
| 35 | Hypothetical protein | - | - | - | - | - |
| 36 | Pseudogene (F-box protein-like) | - | - | - | IPR001810  IPR006527  IPR011043  SSF81383 | Cyclin-like F-box  F-box associated type 1  Galactose oxidase, central  F-box domain |
| 37 | F-box protein | - | - | - | IPR001810  IPR006527  IPR011043 | Cyclin-like F-box  F-box associated type 1  Galactose oxidase, central |
| 38 | TCP transcription factor | - | - | - | IPR005333 | TCP transcription factor |
| 39 | Protein of unknown function | - | - | - | IPR006852 | Protein of unknown function DUF616 |
| 40 | Retrotransposon | P10978 | Retrovirus-related Pol polyprotein from transposon TNT 1-94  [Contains: Protease ; Reverse transcriptase ; Endonuclease] | 1e-78 | IPR001584  IPR012337  IPR013103  PTHR11439  SSF56672 | Integrase, catalytic region  Polynucleotidyl transferase, Ribonuclease H fold  Reverse transcriptase, RNA-dependent DNA polymerase  GAG-POL-RELATED RETROTRANSPOSON  DNA/RNA polymerases |
| 41 | Protein of unknown function | - | - | - | IPR007205  IPR007206  IPR011989 | Protein of unknown function DUF383  Protein of unknown function DUF384  Armadillo-like helical |
| 42 | Origin recognition complex subunit 6 | Q9ZVH3 | Origin recognition complex subunit 6 | e-104 | IPR008721 | Origin recognition complex subunit 6 |
| 43 | Sterol desaturase | P38992 | Protein SUR2 (Syringomycin response protein 2) | 2e-50 | IPR001453  IPR006087  IPR006088  PTHR11863 | Molybdopterin binding domain  SUR2-type hydroxylase/desaturase, catalytic region  Sterol desaturase  STEROL DESATURASE |
| 44 | Disease resistance protein | Q8W1E0 | Late blight resistance protein R1-A (Protein R1) | 0 | IPR000767  IPR002182  IPR006121 | Disease resistance protein  NB-ARC  Heavy metal transport/detoxification protein |
| 45 | Disease resistance protein | Q8W1E0 | Late blight resistance protein R1-A (Protein R1) | 0 | IPR000767  IPR002182  PTHR13779  SSF46785  SSF52058  SSF52540 | Disease resistance protein  NB-ARC  HOLLIDAY JUNCTION DNA HELICASE RUVB-RELATED  L domain-like  P-loop containing nucleoside triphosphate hydrolases |
| 46 | Disease resistance protein | Q8W1E0 | Late blight resistance protein R1-A (Protein R1) | 0 | IPR000038  IPR000767  IPR002182 | Cell division/GTP binding protein  Disease resistance protein  NB-ARC |
| 47 | HVA22-like protein | Q07764 | Protein HVA22 | 1e-20 | IPR004345 | TB2/DP1 and HVA22 related protein |
| 48 | Regulator of chromosome condensation, RCC1 | O95714 | HECT domain and RCC1-like domain-containing protein 2 | 7e-47 | IPR000306  IPR000408  IPR009091  IPR011011  IPR011993  PTHR22870  SSF50729 | Zinc finger, FYVE-type  Regulator of chromosome condensation, RCC1  Regulator of chromosome condensation/beta-lactamase-inhibitor protein II  Zinc finger, FYVE/PHD-type  Pleckstrin homology-type  REGULATOR OF CHROMOSOME CONDENSATION  PH domain-like |
| 49 | Retrotransposon | P10978 | POLX_TOBAC Retrovirus-related Pol polyprotein from transposon TNT 1-94  [Contains: Protease ; Reverse transcriptase ; Endonuclease] | e-142 | IPR001584  IPR001878  IPR009007 | Integrase, catalytic region  Zinc finger, CCHC-type  Peptidase aspartic, catalytic |
| 50 | Hypothetical protein | - | - | - | - | - |
| 51 | Transposon-like | - | - | - | - | - |
| 52 | Disease resistance protein | Q8W1E0 | Late blight resistance protein R1-A (Protein R1) | 0 | IPR000767  IPR002182  IPR006121  SSF46785  SSF51695  SSF52047  SSF52540 | Disease resistance protein  NB-ARC  Heavy metal transport/detoxification protein  "Winged helix" DNA-binding domain  PLC-like phosphodiesterases  RNI-like  P-loop containing nucleoside triphosphate hydrolases |
| 53 | Retrotransposon | P10978 | Retrovirus-related Pol polyprotein from transposon TNT 1-94  [Contains: Protease ; Reverse transcriptase ; Endonuclease] | e-114 | IPR001584  IPR001878  IPR009007  IPR012337  IPR013103  PTHR11439  SSF56672  SSF57756 | Integrase, catalytic region  Zinc finger, CCHC-type  Peptidase aspartic, catalytic  Polynucleotidyl transferase, Ribonuclease H fold  Reverse transcriptase, RNA-dependent DNA polymerase  GAG-POL-RELATED RETROTRANSPOSON  DNA/RNA polymerases  Retrovirus zinc finger-like domains |
| 54 | Disease resistance protein | Q8W1E0 | Late blight resistance protein R1-A (Protein R1 | 0 | IPR000767  IPR001611  IPR002182  IPR006121  SSF46785  SSF52047  SSF52540 | Disease resistance protein  Leucine-rich repeat  NB-ARC  Heavy metal transport/detoxification protein  "Winged helix" DNA-binding domain  RNI-like  P-loop containing nucleoside triphosphate hydrolases |
