# Supplementary material for: Comparative sequence analysis of Solanum and Arabidopsis in a hot spot for pathogen resistance on potato chromosome V reveals a patchwork of conserved and rapidly evolving genome segments
Source: BMC Genomics. 2007 May 2;8:112. doi: 10.1186/1471-2164-8-112 (PMC3225836; doi:10.1186/1471-2164-8-112)
Supplement: Additional File 6 — Table S2: Putative orthologs from R1-, r1-contig and haplotypes A, B and C of S. demissum and the corresponding accession numbers are shown. Putative pseudogenes are indicated by "PS". The identities between transposons (TP) are not shown. [file 1471-2164-8-112-S6.doc]

Table S2. Putative orthologs from R1-, r1-contig and haplotypes A, B and C of *S. demissum* and the corresponding accession numbers are shown. Putative pseudogenes are indicated by “PS”. The identities between transposons (TP) are not shown
