# Supplementary figures and images for: Comparative sequence analysis of Solanum and Arabidopsis in a hot spot for pathogen resistance on potato chromosome V reveals a patchwork of conserved and rapidly evolving genome segments
Source: BMC Genomics. 2007 May 2;8:112. doi: 10.1186/1471-2164-8-112 (PMC3225836; doi:10.1186/1471-2164-8-112)

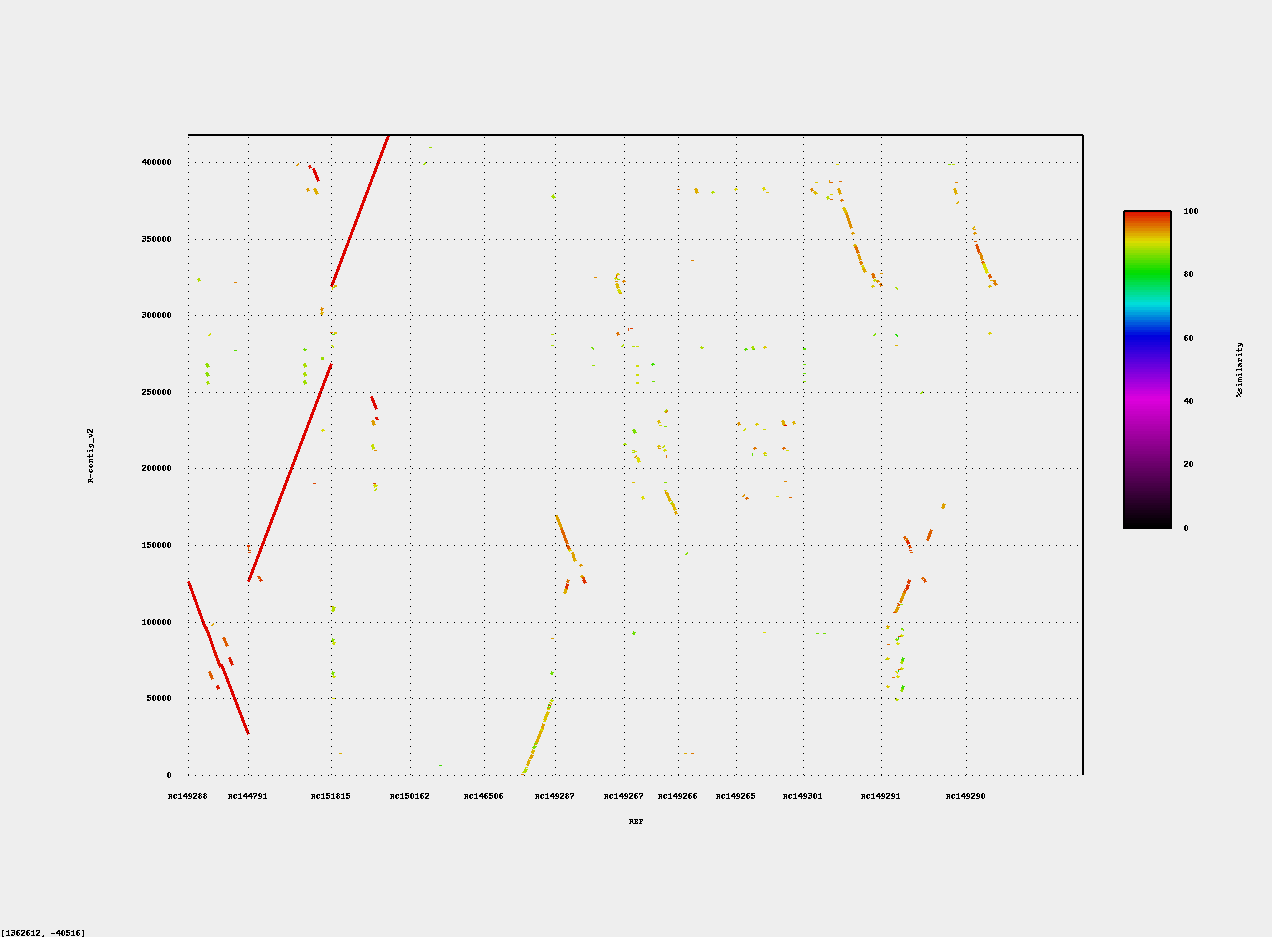

Supplement: Additional File 3 — Figure S3: Dot-Plot comparison computed by MUMmer of the DNA sequence of the R1-contig and individual BAC sequences of S. demissum. One column represents one BAC sequence of S. demissum. The first three columns (from left to right) are from haplotype A, the next seven from haplotype B and the last two columns from haplotype C. The figure shows high similarity and co-linearity between the R1-contig and haplotype A of S. demissum. The first BAC (PGEC446J10, AC149228) is shown in reverse orientation. The region between 270 and 320 kb on the R1-contig is not covered by the non-overlapping BACs PGEC668E02 (AC144791) and PGEC472P22 (AC151815), so we can estimate the gap between these in haplotype A to be around 50 kb in size, covered by the unsequenced BAC PGEC597C19 according to Kuang et al. Only local and limited similarity and co-linearity is observed between the R1-contig and BACs from the B and C haplotypes. The colour reflects percent sequence similarity, with red denoting close to 100% similarity. [file 1471-2164-8-112-S3.doc]

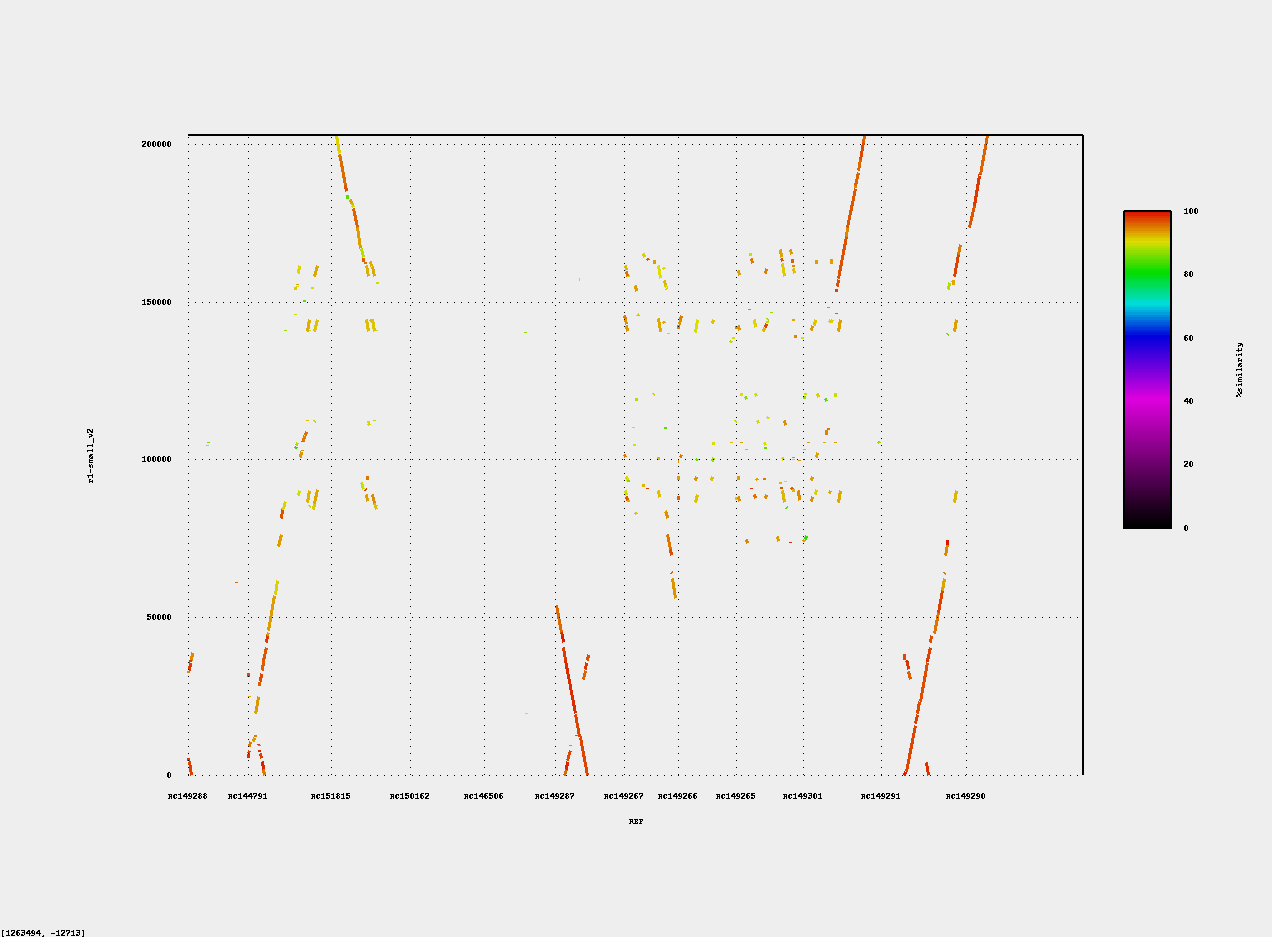

Supplement: Additional File 4 — Figure S4: Dot-Plot comparison computed by MUMmer of the DNA sequence of the r1-contig and BAC sequences of S. demissum. One column represents one BAC sequence of S. demissum. The first three columns (from left to right) are BAC sequences from haplotype A, the next seven from haplotype B and the last two columns from haplotype C. The figure shows two regions of high similarity and co-linearity between the r1-contig and haplotype B and C of S. demissum, with similar but weaker similarity to haplotype A sequences. The first comprises the region between the beginning and 60 kb of the r1-contig and the second runs from 160 kb till the end of the r1-contig. The middle region shows some local similarity that is interrupted by non-conserved sequence. Most of the more significant similarities in this area reflect tandemly duplicated R-genes. The BAC sequences from haplotype C do not contain this region, indicating a deletion in haplotype C as found by Huang et al. An inverted repeat of a RNA-dependent RNA polymerase leads to an X-shaped structure in the plot of the first 40 kb of the r1-contig against the S. demissum sequences, similar to the findings in Figure 2 when comparing to the R1-contig (region B). The colour reflects percent sequence similarity, with red denoting close to 100% similarity. [file 1471-2164-8-112-S4.doc]

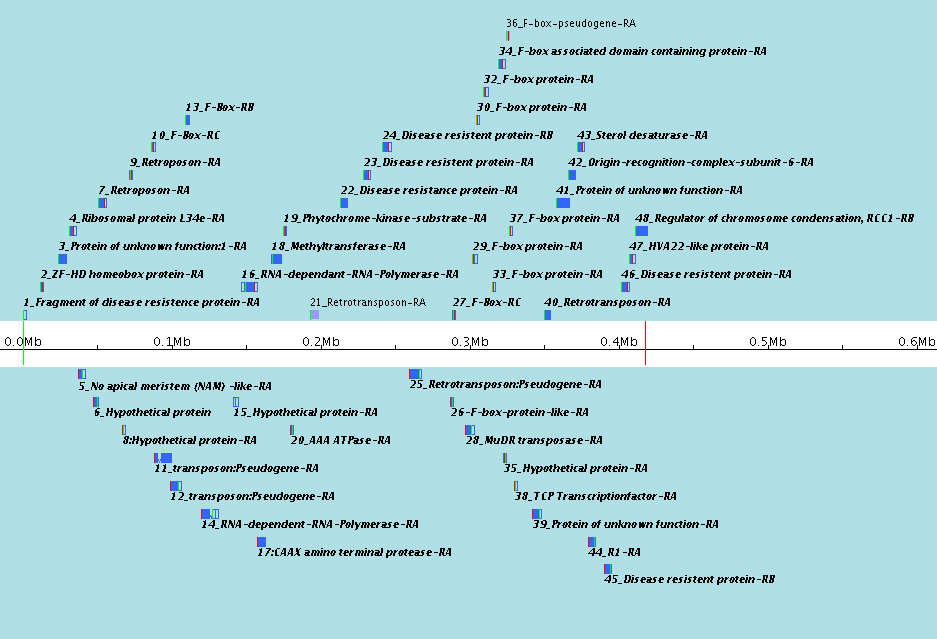

Supplement: Additional File 1 — Figure S1: Gene annotation of R1-contig using APOLLO [file 1471-2164-8-112-S1.png]

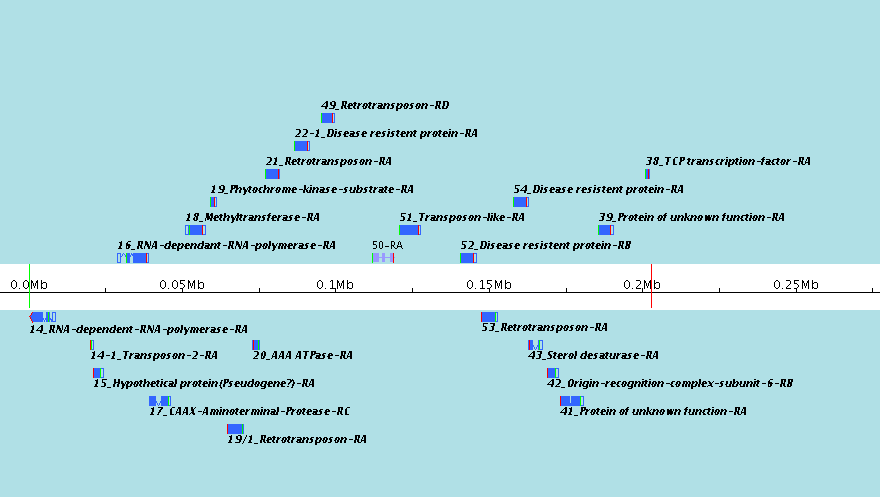

Supplement: Additional File 2 — Figure S2: Gene annotation of r1-contig using APOLLO [file 1471-2164-8-112-S2.png]
